# Does obstructive sleep apnea–induced intermittent hypoxia increase the incidence of solitary pulmonary nodules, thyroid nodules, and other disorders? A retrospective study based on 750 cardiovascular disease patients

**DOI:** 10.1007/s11325-024-03036-x

**Published:** 2024-04-17

**Authors:** Chen Ding, Libo Mao, Yinghong Lu, Sai Wu, Wenyan Ji

**Affiliations:** 1 Shandong University of Traditional Chinese Medicine, Jinan, Shandong China; 2 Department of Cardiology, Qingdao Hiser Hospital Affiliated of Qingdao University (Qingdao Traditional Chinese Medicine Hospital), Qingdao, Shandong China

**Keywords:** Obstructive sleep apnea, Cardiovascular disease, Solitary pulmonary nodules, Thyroid nodules, Intermittent hypoxia

## Abstract

**Background:**

Obstructive sleep apnea (OSA) has been shown to be an important risk factor for cardiovascular disease (CVD), and intermittent hypoxia is an important pathogenetic factor for it. In the clinic, it was found that most CVD patients combined with OSA were also combined with solitary pulmonary nodules (SPN) or thyroid nodules (TN). Are these disorders related to intermittent hypoxia? One study showed that intermittent hypoxia is a pathogenic factor for lung cancer in mice, but there have been no clinical reports. So we conducted a retrospective study to explore whether intermittent hypoxia caused by OSA increases the incidence of SPN, TN, and other disorders.

**Methods:**

We selected 750 patients with cardiovascular disease (CVD), who were divided into the control group and the OSA group according to the result of portable sleep monitoring. Retrospectively analyzed the comorbidities that patients with OSA are prone to and explored the correlation between OSA and those comorbidities.

**Results:**

The incidence of SPN, TN, cervical spondylosis, and carotid-artery plaques was higher in the OSA group than in the control group. These diseases are significantly associated with OSA (*p* < 0.05), and their incidence increased with an elevated apnea–hypopnea index. After excluding interference from age, gender, BMI, smoking history, history of lung disease, and history of tumors, OSA showed a significant correlation with SPN. After excluding age, gender, BMI, and thyroid disease, OSA was associated with TN. Patients with comorbidities have lower nocturnal oxygen saturation and more extended periods of apnea. Logistic multiple regression results revealed that male, advanced age, obesity, CS, and nasal septum deviation were independent risk factors for OSA.

**Conclusions:**

Patients combined with OSA may further develop more comorbidities, such as SPN, TN, and carotid-artery plaques. It may be related to intermittent hypoxia caused by OSA.

## Introduction

Obstructive sleep apnea (OSA) is a prevalent sleep-related breathing disorder characterized by recurrent collapse of the upper airway, respiratory disturbances, apnea, and sleep-wakefulness during sleep, which are manifested by snoring, oppressive wake, daytime somnolence, fatigue, and nocturnal frequency of micturition [1]. Benjamin’s research showed that nearly 1 billion adults aged 30–69 worldwide suffer from OSA, with over 400 million suffering from moderate to severe OSA, even under diagnosis [2]. According to the latest statistics in 2022, China is leading with approximately 176 million OSA patients in the world [3]. Chronic intermittent hypoxia and carbon dioxide retention caused by OSA lead to oxidative stress, systemic inflammation, and sympathetic system activation. OSA is one of the risk factors for cardiovascular disease (CVD) [4], with a prevalence as high as 40 to 80% among CVD patients [3]. Moreover, OSA can induce dysfunction of systems, and is closely related to atherosclerosis, new-onset diabetes, metabolic syndrome, and many other diseases. For this reason, it is crucial to do an in-depth study on OSA individuals.

In the clinic CVD patients, we found that most patients with OSA also suffer from solitary pulmonary nodules (SPN) or thyroid nodules (TN), which are increasingly prevalent. If CVD patients are combined with OSA, they must be in a chronic hypoxic state for a long time. Is intermittent hypoxia an important factor in the occurrence of the above diseases?

The prevalence of SPN and TN shows an increasing trend, with rates of 35.5% and 60%, respectively, in the healthy population. The cause of SPN is complex, including smoking, long-term exposure to polluted environments or heavy dust work, lung infections or malignant tumors, and granulomas or fibrosis or inflammatory lesions caused by other diseases. An animal study showed that intermittent hypoxia was a contributing factor to lung cancer in mice and accelerated tumor growth [5]. However, there have been no clinical reports about it. Regarding TN, only one clinical study has elucidated its correlation with OSA [6].

Accordingly, we retrospectively analyzed the clinical data of 750 patients with CVD to explore whether intermittent hypoxia caused by OSA increases the incidence of the above-mentioned disease.

## Material and methods

### Study participants

The analysis was based on a cohort of patients hospitalized in the cardiovascular department of Qingdao Hiser Hospital Affiliated to Qingdao University from 2021 to 2022. Using the Hospital Information System (HIS), clinical data were collected from 750 patients for analysis, who were aged ≥ 18 years, met at least one cardiovascular disease condition, and had admission records mentioning symptoms such as snoring, apnea, or somnolence.

### Diagnostic criteria

The OSA group (*n* = 454) and the non-OSA group (*n* = 296) were categorized according to the results of portable monitoring (PM). Diagnostic criteria of OSA refer to the International Classification of Sleep Disorders (ICSD-3) issued by the AASM [7]: five or more predominantly obstructive respiratory events (including obstructive and mixed apneas, hypopneas) per hour of sleep during polysomnography (PSG) or out of center sleep testing (OCST) associated with one or more of the following situations: (1) drowsiness during the day, lack of wakefulness after sleep, fatigue, or insomnia, (2) awakening with a gasping or choking sensation, (3) habitual snoring or witnessed apneas, (4) with hypertension, emotional disorder, cognitive impairment, coronary artery disease, stroke, atrial fibrillation, or type 2 diabetes. Or fifteen or more predominantly obstructive respiratory events per hour of PSG or OCST (even in the absence of symptoms). The average number of apnea plus hypopnea events per hour of sleep was represented by the apnea–hypopnea index (AHI).

This study used PM (VentMed Sleep Fairy-A7) instead of PSG, which is considered the gold standard for diagnosis, because in the early stages of the study, most patients were unwilling to use PSG with complex procedures or experienced difficulty falling asleep during use, leading to a decrease in diagnostic rate. Therefore, we adopt a more convenient and comfortable PM. Several comparative studies between PM and PSG have demonstrated a high sensitivity for PM [8]. A domestic study also showed that the diagnostic concordance rate between Sleep Fairy-A7 and PSG was 97.05%, the sensitivity 100%, and the specificity 76.92% [9]. AASM believes that PM can still use the same diagnostic criteria as PSG, but needs to comply with the following [10]: (1) assess patients for snoring, suspected sleep-related breathing disorders, or somnolence before testing and (2) medical staff with professional sleep knowledge review the results and (3) exclude patients with severe comorbidities (including moderate to severe pulmonary disease, neuromuscular disease, congestive heart failure) and other sleep disorders (including central sleep apnea, parasomnias, circadian rhythm disorders). Meanwhile, this study also excluded patients with anatomical abnormalities of the upper airway such as nasal tumors, oropharyngeal tumors, mandibular retraction, and micrognathia; pregnant or lactating women; patients with acute infections, infectious diseases, and prolonged use of medications such as sleeping sedatives. We rejected the data from patients with more than 20% missing clinical information.

This study was approved by the Ethics Committee of the Qingdao Hiser Hospital Affiliated to Qingdao University (approval no. 2023HC08LS002). Ethical principles and applicable laws were strictly adhered to in conducting the study and informed consent was obtained from the participants.

### Basic information collection

Baseline demographic data (gender, age, body mass index [BMI], smoking history) and relevant past history (coronary heart disease, hypertension, heart failure, dilated cardiomyopathy, arrhythmia, diabetes mellitus, cerebral infarction, and cancer) were collected by using HIS.

### Sleep study

According to the sleep monitoring report, we recorded apnea–hypopnea index (AHI), lowest oxygen saturation (LSpO_2_), mean oxygen saturation (MSpO_2_), total apnea time (TAT), longest time of apnea (LAT), hypoventilation counts (H counts), and obstructive sleep apnea counts (OSA counts).

### Laboratory examinations

We selected 450 patients for whom laboratory test results were available and recorded values of red blood cell count (RBC), hematocrit (HCT), mean corpuscular hemoglobin concentration (MCHC), mean corpuscular volume (MCV), low-density lipoprotein cholesterol (LDL-C), and uric acid (UA).

### Imaging information

Patients were documented for SPN based on lung CT results; cervical spondylosis (CS) based on cervical spine CT or MRI results; cerebral infarction (CI) based on brain CT results; TN based on thyroid ultrasound results; and carotid-artery plaques (CAP) based on carotid vascular ultrasound results.

### Statistical methods

For statistical analyses, the SPSS 25.0 program was employed. Descriptive statistics were used for general information, with measurement data expressed as mean ± standard deviation (x ± s) and count data expressed as a percentage (%). Independent samples *t*-test was used for comparison between OSA and non-OSA groups, and the chi-square test was used for comparison of rates. The relationship between OSA and comorbidities and between comorbidities and various sleep apnea monitoring indicators was analyzed using binary logistic stepwise regression. Risk factors for CVD combined with OSA were analyzed using multifactorial regression. *p* values ≤ 0.05 were considered to be statistically significant.

## Results

### Demographics and clinical characteristics of patients

**Fig. 1:**
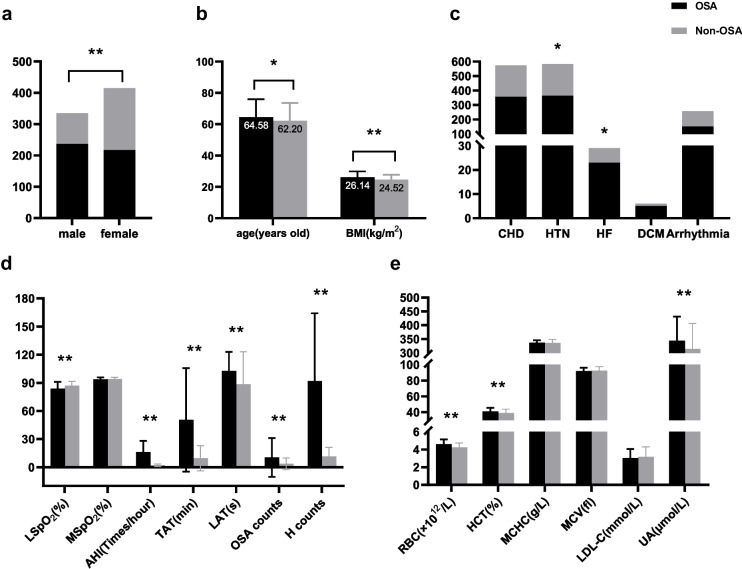
Comparison of the basic conditions between two groups. **a** Differences in gender. **b** Differences in age and BMI. **c** Differences in five kinds of CVDs. **d** Differences in sleep monitoring indicators. **e** Differences in laboratory tests. BMI, body mass index; CHD, coronary heart disease; HTN, hypertension; HF, heart failure; DCM, dilated cardiomyopathy; AHI, apnea–hypopnea index; LSpO_2_, lowest oxygen saturation; MSpO_2_, mean oxygen saturation; TAT, total apnea time; LAT, longest time of apnea; H counts, hypoventilation counts; OSA counts, obstructive sleep apnea counts; RBC, red blood cell count; HCT, hematocrit; MCHC, mean corpuscular hemoglobin concentration; MCV, mean corpuscular volume; LDL-C, low-density lipoprotein cholesterol; UA, uric acid. *p ≤ 0.05, **p ≤ 0.001

In the initial stage of the statistical study, we contrasted the differences in several parameters between the two patient groups. Compared with the non-OSA group, the OSA group contained significantly more male than female patients (Fig. 1a), as well as significantly higher age and BMI (Fig. 1b). It was noted that while there were substantial statistical differences in hypertension and heart failure between the two groups, there were no significant statistical differences in coronary heart disease, dilated cardiomyopathy, or arrhythmia (Fig. 1c). All the sleep monitoring indicators except MSpO_2_ were significantly different between the two groups (Fig. 1d), which means AHI, LSpO_2_, TAT, LAT, H counts, and OSA counts had practical significance for the OSA patients in this study and can be further explored for correlation analysis. Regarding laboratory tests, it is observed that the levels of RBC, HCT, and UA in the OSA group were significantly higher than those in the control group (Fig. 1e).

### Relationship and correlation between OSA and comorbidities

**Fig. 2:**
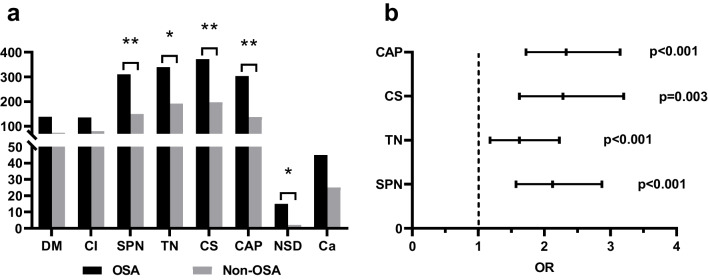
Relationship and correlation between OSA and comorbidities. **a** Differences in comorbidities between the two groups. **b** The correlation of differential comorbidities with OSA. DM, diabetes mellitus; CI, cerebral infarction; SPN, solitary pulmonary nodules; TN, thyroid nodules; CS, cervical spondylosis; CAP, carotid-artery plaque; NSD, nasal septum deviation; Ca, cancer; OR, odds ratio. *p ≤ 0.05, **p ≤ 0.001

In order to explore the correlation between OSA and comorbidities, we first analyzed the differences in common clinical comorbidities between the two groups (Fig. 2a). The rates of SPN, TN, CS, CAP, and nasal septum deviation (NSD) were considerably greater in the OSA group compared to the non-OSA group, whereas DM, CI, and Ca did not differ in significance. Secondly, utilizing “occurrence of OSA” as the independent variable and “comorbidities with significant differences in the chi-square test (SPN, TN, CS, CAP)” as the dependent variable, the results of binary logistic regression studies were statistically significant (Fig. 2b). The results indicate that OSA is correlated with SPN, TN, CS, and CAP, respectively, after adjusting gender, age, related vascular disease, diabetes, and other factors (Table 1).

**Table 1:** Hierarchical regression analysis of OSA with SPN, TN, CS, CAP

Comorbidities	Value	Model 1	Model 2	*P*
SPN	*R*^2^	0.017	0.045	*p* < 0.001
	Δ*R*^2^	0.017	0.028	
	*F*	12.633	21.995	
TN	*R*^2^	0.038	0.051	*P* = 0.002
	Δ*R*^2^	0.038	0.013	
	*F*	14.810	10.057	
CS	*R*^2^	0.009	0.042	*p* < 0.001
	Δ*R*^2^	0.009	0.033	
	*F*	7.116	25.719	
CAP	*R*^2^	0.205	0.221	*p* < 0.001
	Δ*R*^2^	0.205	0.016	
	*F*	63.998	15.660	

*SPN* solitary pulmonary nodules, *TN* thyroid nodules, *CS* cervical spondylosis, *CAP* carotid-artery plaque, *R*^2^ coefficient of determination, *F* variance ratio

We used SPN as the dependent variable, OSA as the independent variable, and gender, age, BMI, smoking history, lung disease history, and tumor history as control variables for correlation analysis. The results showed that after excluding the aforementioned control variables, there was still a strong correlation between OSA and SPN (Table 2). Similarly, we used TN as the dependent variable, OSA as the independent variable, and gender, age, BMI, and thyroid disease history as control variables for correlation analysis. OSA was correlated with TN after excluding these control variables (Table 3).

**Table 2:** Correlation analysis between OSA and SPN

Model			Unstandardized coefficient		Standard coefficient	*t*	Sig	VIF
			*B*	Standard error	Beta			
(Constant)			0.286	0.169		1.692	0.091	
IV	OSA		0.176	0.038	0.177	4.699	< 0.001	1.105
	Non-OSA		0					
CV	Age		0.004	0.002	0.100	2.729	0.007	1.056
	BMI		− 0.002	0.005	− 0.014	− 0.383	0.702	1.084
	Gender	Male	− 0.177	0.109	− 0.181	− 1.626	0.104	9.667
		Female	0					
	Smoking history	Yes	0.158	0.109	0.161	1.448	0.148	9.633
		No	0					
	Lung disease	Yes	0.034	0.053	0.023	0.640	0.532	1.034
		No	0					
	Tumor history	Yes	0.024	0.061	0.014	0.388	0.698	1.020
		No	0					
*F*			5.500					
*P*			< 0.001					

*IV* independent variable, *CV* control variable, *F* variance ratio, *VIF* variance inflation factor

**Table 3:** Correlation analysis between OSA and TN

Model			Unstandardized coefficient		Standard coefficient	*t*	Sig	VIF
			*B*	Standard error	Beta			
(Constant)			0.000	0.159		0.001	0.999	
IV	OSA		0.097	0.035	0.104	2.769	0.006	0.908
	Non-OSA		0					
CV	Age		0.006	0.001	0.161	4.406	< 0.001	1.050
	BMI		0.007	0.005	0.055	1.494	0.136	1.081
	Gender	Female	0.113	0.034	0.123	3.357	0.001	1.063
		Male	0					
	Thyroid disease	Yes	0.009	0.057	0.005	0.152	0.879	1.031
		No	0					
*F*			8.465					
*P*			< 0.001					

*IV* independent variable, *CV* control variable, *F* variance ratio, *VIF* variance inflation factor

### Correlation between comorbidities and sleep monitoring indicators

**Fig. 3:**
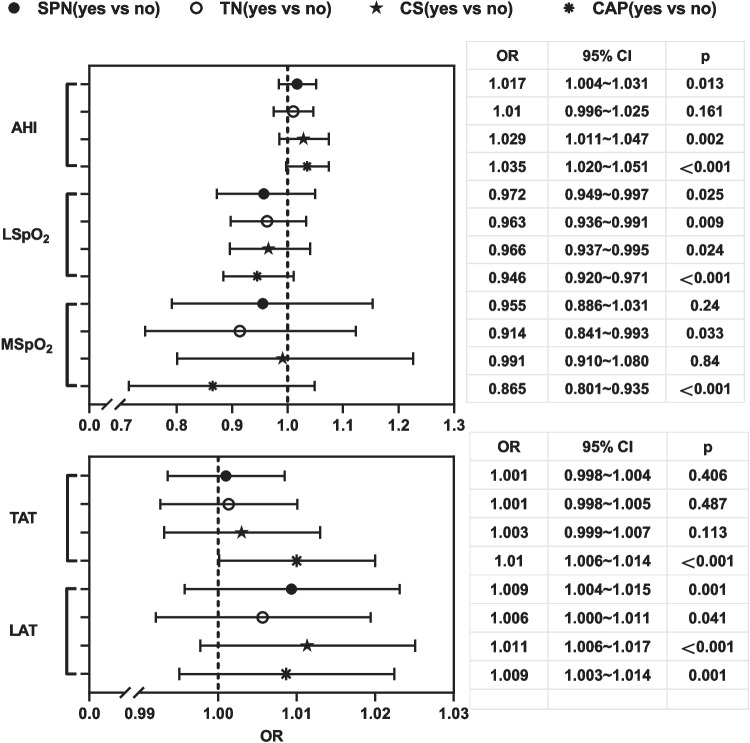
Correlation between comorbidities and sleep monitoring indicators. AHI, apnea–hypopnea index; LSpO_2_, lowest oxygen saturation; MSpO_2_, mean oxygen saturation; TAT, total apnea time; LAT, longest time of apnea; CI, confidence interval; OR, odds ratio

To explore the mechanism by which OSA leads to comorbidities, we respectively performed binary logistic regression analyses of SPN, TN, CS, and CAP (as the dependent variable) with AHI, LSpO_2_, MSpO_2_, TAT, and LAT (as the independent variable) (Fig. 3). The results revealed that AHI was independently associated with SPN (*P* = 0.013), CS (*P* = 0.002), and CAP (*P* < 0.001) (all odds ratio [OR] > 1), which points out that the higher the severity of OSA, the higher the incidence of these three diseases. Besides, SPN, TN, CS, and CAP were significantly associated with LSpO_2_ and LAT (all *P* < 0.05), and their incidence was negatively correlated with LSpO_2_ (OR < 1) and positively correlated with LAT (OR > 1). It is suggested that the lower the LSpO_2_ and the higher the LAT, the more likely it is that the four diseases mentioned above will occur.

### Males, high BMI, cervical spondylosis, and nasal septum deviation were risk factors for OSA

**Fig. 4:**
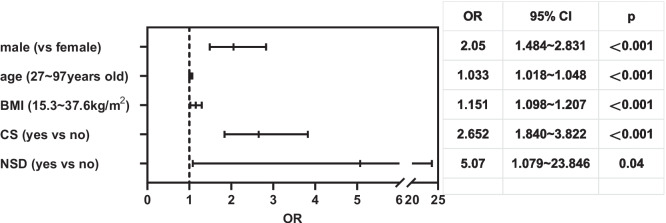
Multiple regression analysis of risk factors for OSA. BMI, body mass index; CS, cervical spondylosis; NSD, nasal septum deviation; CI, confidence interval; OR, odds ratio

We used gender, age, BMI, CS, NSD, and other possible risk factors for OSA as independent variables, and “occurrence of OSA” as the dependent variable. Multiple regression analysis demonstrated that males, high BMI, CS, and NSD were independently associated with the occurrence of OSA (Fig. 4).

## Discussion

### Intermittent hypoxia caused by OSA

With the improvement of people’s living standards and changes in lifestyle, the incidence of OSA has increased year by year. Intermittent hypoxia due to OSA can cause a number of pathophysiologic changes [11] such as oxidative stress [12], systemic inflammation [13], and sympathetic system activation [14], which are the pathological basis for the many diseases. Thus, intermittent hypoxia is an important part of disease development and should not be ignored.

### Solitary pulmonary nodules and thyroid nodules

Figure 2 shows a positive correlation between OSA and SPN (*P* < 0.001, OR > 1), indicating that the presence of OSA may lead to an increase in the incidence of SPN. However, there were many confounding factors among them. A prospective cohort study [15] showed that age, smoking history, lung disease history, occupational exposure, and family history of cancer may affect the incidence of pulmonary nodules. Sun Ying [16] found a correlation between age, respiratory system diseases, BMI, and the incidence of pulmonary nodules through a study of 69,957 healthy individuals. Chen Liqun’s research [17] also found that individuals with a history of tumors are more likely to detect pulmonary nodules. Therefore, we excluded some variables related to SPN (age, gender, BMI, smoking history, history of lung disease, history of tumors) and found that the results were still significant (Table 2). Similarly, some studies have confirmed an independent correlation between women, age, BMI, metabolic syndrome, thyrotoxicosis, and TN [18–20]. So we found a correlation between OSA and TN after excluding age, gender, BMI, and thyroid disease (Table 3). We speculate that OSA may be one of the reasons for the occurrence of SPN and TN. Because we have not comprehensively excluded all confounding factors and there are limited reports on OSA and two diseases, this is only our speculation and further investigation is expected in the future. This study also conducted a correlation analysis between SPN, TN, and some sleep indicators (AHI, LSpO_2_, MSpO_2_, TAT, LAT). We found that patients with SPN or TN have lower nighttime blood oxygen concentration and longer apnea time (Fig. 3). Intermittent hypoxia may be a pathological factor in the process of SPN or TN caused by OSA. This is just speculation, and we hope to have relevant pathological research in the future.

### Carotid-artery plaques

The development of OSA is accompanied by a degree of hypoxia, which results in enhanced lipid loading in foam cells and susceptibility of endothelial cells to free radical damage, leading to the formation of atherosclerotic plaques. This study confirms that there is a significant correlation between CAP and OSA, and the incidence of the former increased with the severity of the latter. CVD patients generally exhibit atherosclerosis, which inevitably leads to a high incidence of CAP in this research. However, the result was still statistical after adjusting gender, age, related vascular disease, diabetes, and other factors (Table 1). This agrees with much recent research [21–23].

### Abnormal metabolism

It has been confirmed that OSA can cause abnormal lipid metabolism [24]. On the one hand, recurrent intermittent hypoxia during sleep results in elevated levels of hypoxia-inducible factor-1. Lipolysis of adipose tissue and increased biosynthesis in the liver promote LDL-C secretion [25]. A significant correlation between LDL-C and OSA has been shown in numerous studies. However, this study could not exclude the fact that some patients were required to take lipid-lowering drugs for their condition, which had an impact on the results. On the other hand, hyperlipidemia has been implicated as a mechanism linking OSA and coronary heart disease [26]. OSA-induced dyslipidemia worsens the cardiac coronary conditions in CVD patients.

The data showed significant differences in the RBC, HCT, and UA levels between the two groups, which were related to chronic intermittent hypoxia leading to a compensatory response, suggesting that OSA may cause varying degrees of metabolic syndrome [27]. Furthermore, elevated hematocrit may affect hemorheology and lead to hypercoagulation of blood, exacerbating cardiovascular disease. To sum up, the presence of OSA in patients with CVD can lead to metabolic abnormalities, and OSA should be actively improved to avoid disease progression.

### Prevention and treatment of OSA

Upper airway collapse is the main pathologic feature of OSA, while age, gender, obesity, nasopharyngeal diseases, and CS are independent risk factors for OSA [28–31]. In patients with OSA, the prevalence is significantly higher in males than in females, and the prevalence increases with age. In addition, obese patients with submucosal fat deposits in the oropharynx are prone to apnea. Nasal disorders such as rhinopolypus, turbinate hypertrophy, NSD, and chronic rhinitis result in obstruction of nocturnal nasal breathing, making the pressure in the pharyngeal cavity lower than the atmospheric pressure, and then increased pharyngeal transmural pressure leads to airway closure. When nasal obstruction triggers mouth breathing, it increases the incidence of collapse of the lateral pharyngeal wall and tongue root [32, 33], resulting in apnea and hypoventilation. Furthermore, changes in modern lifestyles have exacerbated the occurrence of CS. OSA occurs when a cervical disc herniation or cervical degeneration causes increased compression of the tissues surrounding the pharyngeal airway space, resulting in pharyngeal stenosis [34]. This is consistent with the anatomical impact of cervical morphologic changes on the pharyngeal airway in patients with CS [35]. So, OSA can be avoided to some extent by weight loss, regular and adequate sleep, and improvement of cervical spine morphology. Patients with nasopharyngeal disorders should be motivated to undergo corrective or surgical procedures that will improve ventilation. Continue positive airway pressure (CPAP) is the first-line treatment for patients with OSA, which improves nocturnal hypoventilation and reduces the risk of CVD and multi-organ damage [36]. In conclusion, intermittent hypoxia is worthy of attention. Improving ventilation and correcting intermittent hypoxia are the main methods for treating OSA. This may improve many diseases caused by OSA.

### Limitations

Several limitations of this study should be considered. First, as this is a retrospective study, it is not possible to exclude the influence of some patients taking relevant medications due to their condition on the results of the study. Second, this research was based on patients with CVD, and we hypothesize that OSA can still increase the incidence of SPN, TN, and other diseases in the general population. Therefore, we will conduct a large-scale prospective study in the population in the future to further characterize the correlation between OSA and SPN and TN, as well as the mechanisms, on the basis of excluding relevant risk factors.

## Conclusion

To summarize, patients combined with OSA may further develop more comorbidities, such as SPN, TN, and CAP. After excluding interference from age, gender, BMI, smoking history, history of lung disease, and history of tumors, OSA still showed a significant correlation with SPN. After excluding age, gender, BMI, and thyroid disease, OSA was still associated with TN. Patients with SPN or TN have lower nighttime blood oxygen concentration and longer apnea time, which means these two diseases may be related to intermittent hypoxia caused by OSA. A detailed investigation into OSA and SPN or TN should be further conducted in the future.

## Data Availability

The data that support the findings of this study are available from the authors.
